# Suppression of B function by chimeric repressor gene-silencing technology (CRES-T) reduces the petaloid tepal identity in transgenic *Lilium* sp.

**DOI:** 10.1371/journal.pone.0237176

**Published:** 2020-08-03

**Authors:** Masahiro Otani, Kaiki Aoyagi, Masaru Nakano

**Affiliations:** Faculty of Agriculture, Niigata University, Nishi-ku, Niigata, Japan; University of Tsukuba, JAPAN

## Abstract

Some monocotyledonous plants, including liliaceous, amaryllidaceous and iridaceous ones, produce flowers with petaloid tepals in whorls 1 and 2 organs. For explaining the molecular mechanism of two-layered petaloid tepal development, the modified ABC model has been proposed, in which B class genes are expressed in whorl 1 organs as well as in whorls 2 and 3 organs. We have previously obtained results strongly support the modified ABC model by chimeric repressor gene-silencing technology (CRES-T)-mediated suppression of B function in the liliaceous plant *Tricyrtis* sp. In the present study, we introduced a CRES-T construct derived from the B class gene of *Tricyrtis* sp. (*TrihDEFa-SRDX*) into *Lilium* sp. in order to examine the effect of suppressing B function on the floral organ identity. Flowers of transgenic plants did not open fully and had pale pink-colored tepals with decreased numbers of papillae on the adaxial side in whorls 1 and 2 compared with those of non-transgenic plants. No apparent morphological alterations were observed in whorls 3 and 4 organs. Both the amount of total anthocyanins and the expression levels of endogenous flavonoid biosynthesis-related genes (*LhMYB12*, *LhbHLH2*, *LhCHS*, *LhF3H*, *LhF3’H*, *LhDFR* and *LhANS*) decreased in whorls 1 and 2 organs of transgenic plants compared with non-transgenic plants. In addition, the expression levels of endogenous B class genes (*LFDEF*, *LFGLOA* and *LFGLOB*) decreased in transgenic plants and the level was negatively correlated with the degree of morphological alteration. Thus suppression of B function may reduce the identity of petaloid tepals in whorls 1 and 2 of transgenic *Lilium* sp.

## Introduction

Molecular mechanisms of floral organ development have been well understood by intensive studies using eudicotyledonous model plants such as *Arabidopsis thaliana* and *Antirrhinum majus*. The ABC model represents the relationship between floral organ development and expression patterns of three classes of floral homeotic genes, A, B, and C class genes [1, 2]. According to this model, B class gene specifies petal formation in combination with A class gene in whorl 2, and stamen formation in combination with C class gene in whorl 3. B class genes comprise two paralogous genes, *DEFICIENS* (*DEF*)/*APETALA3* (*AP3*) and *GLOBOSA* (*GLO*)/*PISTILATA* (*PI*), and DEF/AP3 and GLO/PI proteins interact directly to form functional complexes [3, 4]. In various eudicotyledonous plants, mutation or suppression of B class genes has resulted in the conversion of petals into sepal-like organs and stamens into pistil-like organs [1, 5–12].

In contrast to eudicotyledonous plants, some monocotyledonous plants, including liliaceous, amaryllidaceous and iridaceous ones, produce flowers with two-layered petaloid tepals in whorls 1 and 2. In order to explain the molecular mechanism of two-layered petaloid tepal development, the modified ABC model has been proposed [13]. According this model, expression of B class genes extends to whorl 1 in addition to whorls 2 and 3. The modified ABC model has been supported indirectly by expression analysis of B class genes in various plant species with two-layered petaloid tepals such as *Tulipa gesneriana* [14, 15], *Agapanthus praecox* [16], *Muscari armeniacum* [17, 18], *Dendrobium crumenatum* [19], *Phalaenopsis aphrodite* [20], *Crocus sativus* [21] and *Alstroemeria ligtu* [22]. Recently, we have obtained results directly supporting the modified ABC model in the liliaceous plant *Tricyrtis* sp. [23] by chimeric repressor gene-silencing technology (CRES-T), which suppresses target genes of a transcription factor dominantly by expressing a fusion protein of the transcription factor with a repression domain [24]. Transgenic *Tricyrtis* sp. plants with suppressed B function by CRES-T produced sepaloid-tepals instead of petaloid tepals in whorls 1 and 2, and pistil-like organs instead of stamens in whorl 3. However, effect of suppressing B function on the floral organ development is still unclear in other plant species with two-layered petaloid tepals.

*Lilium* spp. (Liliaceae) are one of the most popular plants with two-layered petaloid tepals. There have been some reports in *Lilium* spp. that expression of B class genes extends to whorl 1 in addition to whorls 2 and 3 [25–27]. Although these reports support the modified the ABC model, no direct evidence by suppression or mutation of B class genes has been obtained in *Lilium* spp. yet. *Lilium* spp. have some advantages for studying floral organ development as follows: (1) an efficient and reproducible system of *Agrobacterium*-mediated genetic transformation has been established [28]; (2) they have large floral organs compared with other plant species with two-layered petaloid tepals; and (3) several major genes involving in floral organ development, such as *APETALA1*/*SQUAMOSA*-, *DEF*/*AP3*-, *GLO*/*PI*- and *AGAMOUS*-like genes, have so far been isolated [26, 27, 29–31].

In the present study, we produced and characterized transgenic plants of an oriental hybrid lily, *Lilium* cv. Acapulco, carrying a CRES-T construct derived from the B class gene of *Tricyrtis* sp. in order to examine the effect of suppressing B function on the floral organ identity.

## Materials and methods

### Plant material and production of transgenic plants

Potted plants of *Lilium* cv. Acapulco were cultivated in a greenhouse without heating.

*Agrobacterium tumefaciens* strain EHA101/pBCSH-CrB was used for transformation. Full-length coding region of the B class gene of *Tricyrtis* sp. (*TrihDEFa*) was fused with the ERF-associated amphiphilic repression (EAR) motif repression domain (*SRDX*) [32], resulting in *TrihDEFa*-*SRDX*. T-DNA region of the binary vector pBCSH-CrB contained *TrihDEFa*-*SRDX* under the control of the cauliflower mosaic virus (CaMV) 35S promoter with a translational enhancer sequence of tobacco mosaic virus (omega enhancer), and the hygromycin phosphotransferase gene (*HPT*) under the control of the nopaline synthase (NOS) promoter as a selectable marker gene (S1 Fig in S1 File).

Inoculation and co-cultivation of filament-derived calli with *Agrobacterium*, selection of transgenic cells and tissues, and regeneration of transgenic plants were performed as previously described [28]. The presence of *HPT* in putative transgenic plants was confirmed by PCR analysis with the primer set hpt290-F and hpt290-R (S1 Table in S1 File).

### Morphological characterization of transgenic plants

Transgenic plants were transplanted to pots and cultivated in a growth chamber. Three years after cultivation, morphological characterization was performed during the flowering season. The total numbers of papillae on the adaxial side of whorls 1 and 2 organs were counted. Scanning electron microscopy (SEM) observation of the surface of whorls 1 and 2 organs was performed as previously described [33]. The size of epidermal cells was measured using ImageJ software [34]. Extraction and measurement of total anthocyanins in whorls 1 and 2 organs were carried out as previously described [35].

### RNA isolation and gene expression analysis

Total RNA was extracted using the RNeasy Plant Mini Kit (QIAGEN, Hilden, Germany) and then treated with the RNase-Free DNase Set (QIAGEN, Hilden, Germany) according to the manufacturer’s instructions. For cDNA synthesis, 500 ng of total RNA was reverse-transcribed using the PrimeScript™ RT reagent Kit (Takara Bio Inc., Shiga, Japan) in accordance with the manufacturer’s instructions.

For detecting endogenous genes for two main transcriptional regulator of the flavonoid biosynthesis [R2R3-type myeloblastosis (*LhMYB12*) and basic helix-loop-helix (*LhbHLH2*)] [36, 37], endogenous genes for five flavonoid biosynthetic enzymes [chalcone synthase (*LhCHS*), flavanone-3-hydroxylase (*LhF3H*), flavonoid 3’-hydroxylase (*LhF3’H*), dihydroflavonol 4-reductase (*LhDFR*) and anthocyanin synthase (*LhANS*)], and the transgene (*TrihDEFa-SRDX*), semi-quantitative RT-PCR was performed using the EmeraldAmp® MAX PCR Master Mix (Takara Bio Inc., Shiga, Japan) on the T100™ Thermal Cycler (Bio-Rad, CA, USA). Amplified products were analyzed by electrophoresis on 1.5% agarose gels. The actin gene of *Lilium* sp. (*LhACT*) was used as an internal control.

Real-time RT-PCR was performed using the SYBR® Premix Ex Taq™ II (Takara Bio Inc., Shiga, Japan) on the MiniOpticon^TM^ Detecter (Bio-Rad, CA, USA) as previously described [23]. The relative amounts of endogenous B class gene (*LFDEF*, *LFGLOA* and *LFGLOB*) transcripts were calculated using the comparative cycle threshold method, and results were normalized to *LhACT*.

Accession number of genes, primer sets and PCR conditions used for these analyses are listed in S1 Table in S1 File.

## Results

### Morphological characterization of transgenic plants

Eighteen independent transgenic plants of *Lilium* cv. Acapulco carrying *TrihDEFa*-*SRDX* were obtained and termed LiCrB (transgenic Lilies with CRES-T construct for B class genes) strains. The presence of the transgene *HPT* was confirmed by PCR analysis (S2 Fig in S1 File). Morphological characterization was performed three years after cultivation in pots during the flowering season.

**Fig 1:**
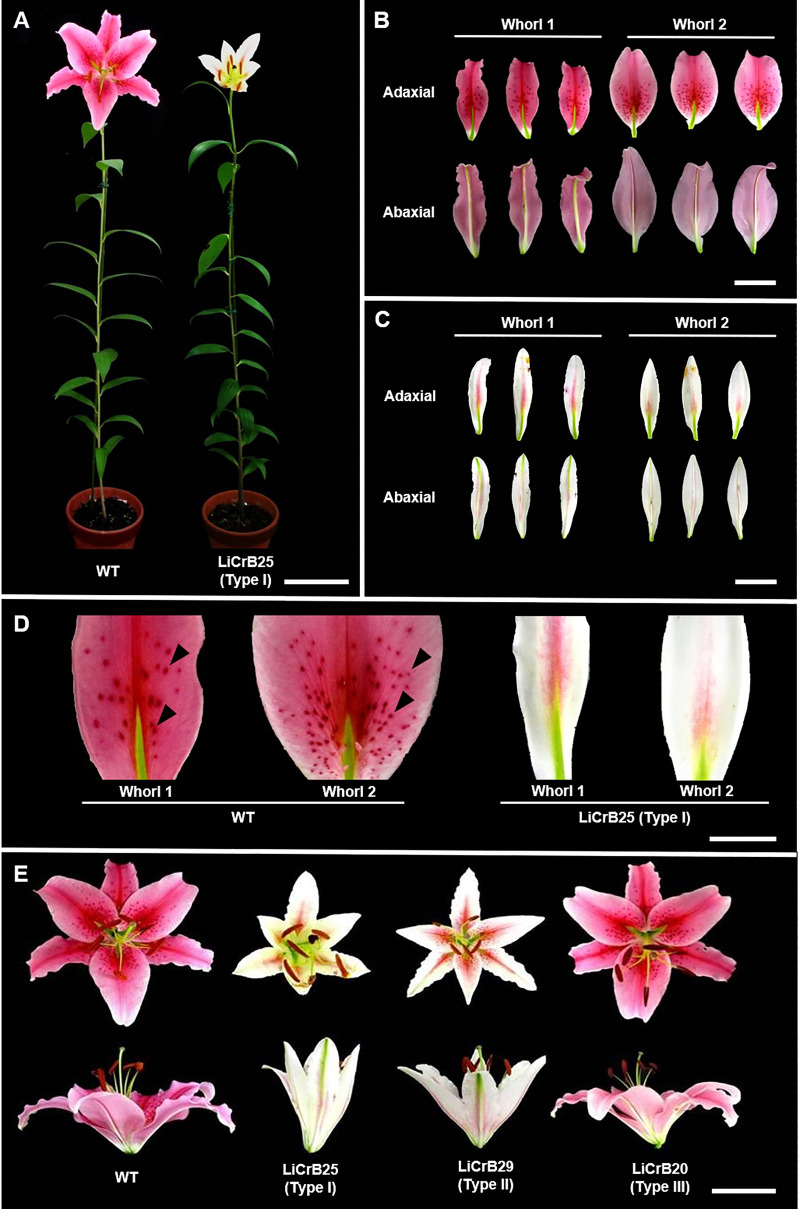
Morphological characterization of the wild-type (WT) and transgenic plants (LiCrBs). Type I (LiCrB25), Type II (LiCrB29) and Type III (LiCrB20) transgenic plants showed severe, moderate and no morphological alterations, respectively. (A) Flowering plants of WT and LiCrB25. Bar = 10 cm. (B, C) Whorls 1 and 2 organs of (B) WT and (C) LiCrB25. Bar = 5 cm. (D) Close-up of the adaxial side of whorls 1 and 2 organs of wild-type (WT) and LiCrB25. Black arrowheads indicate papillae. Bar = 2 cm. (E) Flowers of WT, LiCrB25, LiCrB29 and LiCrB20. Bar = 5 cm.

**Fig 2:**
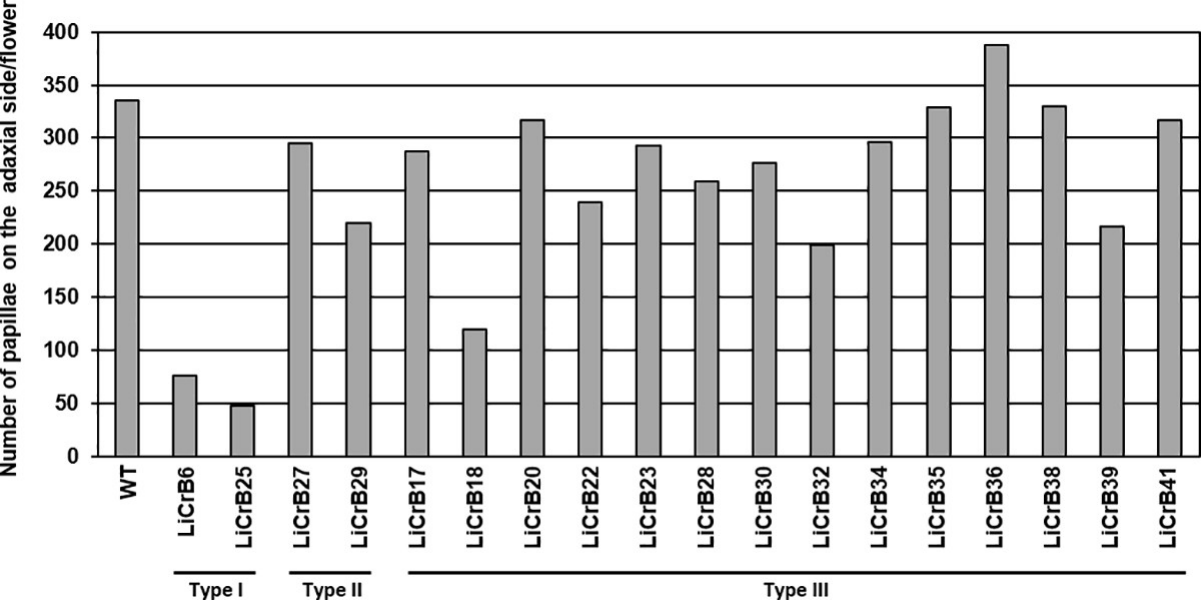
Number of papillae on the adaxial side of whorls 1 and 2 organs of wild-type (WT) and transgenic plants (LiCrBs). Type I, Type II and Type III transgenic plants showed severe, moderate and no morphological alterations, respectively.

In wild-type plants (WT), flowers had deep pink-colored tepals in whorls 1 and 2, and many papillae were formed on the adaxial side of these tepals (more than 300 papillae per flower) (Figs 1 and 2). LiCrB strains could be classified into three types according to the degree of morphological alteration in floral organs. Type I LiCrB strains (LiCrB6 and LiCrB25) showed severe morphological alterations in floral organs (Fig 1; Table 1). These strains produced non-fully-opened flowers (funnel-shaped flowers) with pale pink-colored and narrow tepals in whorls 1 and 2. The number of papillae per flower much decreased in Type I LiCrB strains compared with WT (76 and 48 papillae in LiCrB6 and LiCrB25, respectively) (Figs 1D and 2). Type II LiCrB strains (LiCrB27 and LiCrB29) showed moderate morphological alterations in floral organs (Fig 1E; S3 Fig in S1 File; Table 1). They produced moderately-opened-flowers (cup-shaped flowers) with pale pink-colored and narrow tepals in whorls 1 and 2. In contrast to Type I LiCrB strains, there were no large differences in the number of papillae per flower between Type II LiCrB strains and WT (295 and 220 papillae in LiCrB27 and LiCrB29, respectively) (Fig 2). Fourteen strains showed no apparent morphological alterations in any floral organs, and they were classified into Type III LiCrB strains (Fig 1E; Table 1). All of the LiCrB strains showed no morphological alterations in whorls 3 and 4 organs as well as in vegetative organs (S3 and S4 Figs in S1 File).

**Table 1:** Morphological characterization of wild-type plants and transgenic plants (LiCrBs) during the flowering season.

Plant strain	Flower diameter (cm)	Flower length (cm) ^a^	Flower diameter/length	Pollen fertility ^b^	Whorl 1 organ length (cm) ^c^	Whorl 1 organ width (cm) ^c^	Whorl 2 organ length (cm) ^c^	Whorl 2 organ width (cm) ^c^
Wild-type	20.1	3.6	5.6	91.9±3.0 a	12.5±0.1 a	4.0±0.0 a	12.3±0.1 a	5.6±0.1 a
Type I								
LiCrB6	13.6	10.2	1.3	65.5±6.6 c	10.5±0.2 b	2.7±0.1 de	10.6±0.1 cd	3.2±0.1 c
LiCrB25	9.2	9.9	0.9	53.5±3.6 cd	10.6±0.4 b	2.4±0.1 e	10.4±0.1 d	3.2±0.2 c
Type II								
LiCrB27	15.8	6.8	2.3	46.2±4.1 d	11.1±0.1 b	2.9±0.0 cd	10.9±0.1 bcd	4.1±0.2 b
LiCrB29	13.4	8.5	1.6	37.9±1.9 d	11.0±0.2 b	2.8±0.0 de	10.8±0.2 bcd	3.8±0.3 bc
Type III								
LiCrB20	18.4	3.2	5.8	74.3±1.7 b	11.4±0.3 ab	3.4±0.0 b	11.3±0.2 b	5.2±0.1 a
LiCrB30	19.0	3.8	5.0	78.4±3.5 ab	11.4±0.1 ab	3.4±0.2 bc	11.2±0.1 bc	5.3±0.0 a

^a^ Vertical length from the base to the tip of flowers.

^b^ Pollen fertility was accessed with acetocarmine staining. Values represent the mean ± standard error of five replicates. Means with different letters are significantly different (*P*<0.05 by Turkey-Kramer’s test).

^c^ Middle position of organs was measured. Values represent the mean ± standard error of the longest three organs for each strain. Means with different letters are significantly different (*P*<0.05 by Turkey-Kramer’s test).

**Fig 3:**
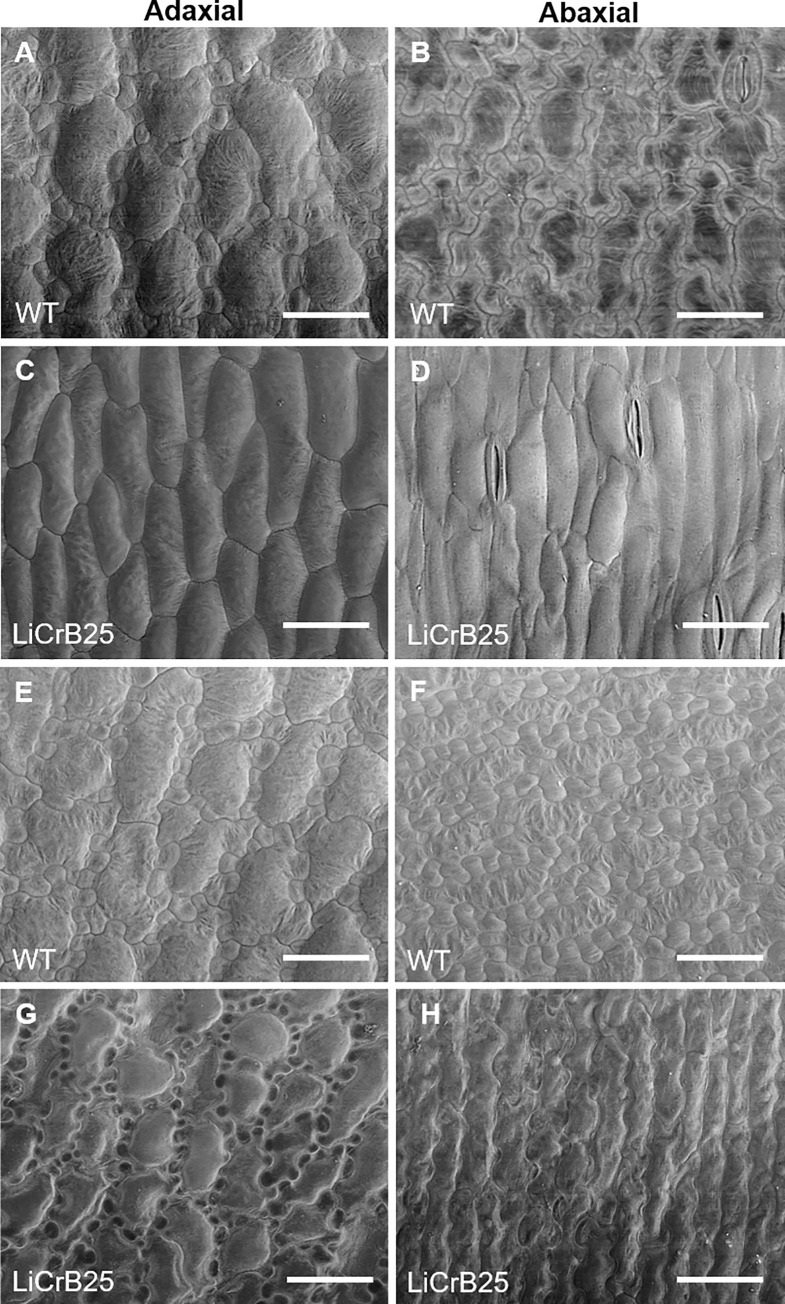
SEM observation of epidermal cells of whorls 1 and 2 organs of wild-type plants (WT) and a Type I transgenic plant showing a severe morphological alteration (LiCrB25). (A, C) Adaxial surface of the middle position of whorl 1 organs of (A) WT and (C) LiCrB25. (B, D) Abaxial surface of the middle position of whorl 1 organs of (B) WT and (D) LiCrB25. (E, G) Adaxial surface of the middle position of whorl 2 organs of (E) WT and (G) LiCrB25. (F, H) Abaxial surface of the middle position of whorl 2 organs of (F) WT and (H) LiCrB25. Bars = 100 μm.

The surfaces of whorls 1 and 2 organs of WT and LiCrB25 (Type I) was observed through SEM. In WT, adaxial and abaxial surfaces of the middle position of whorls 1 and 2 organs mainly consisted of flat and complicated irregular-shaped cells (Fig 3A, 3B, 3E and 3F). On the other hand, adaxial and abaxial surfaces of whorl 1 organs and abaxial surface of whorl 2 organs of LiCrB25 mainly consisted of relatively simple, rectangular cells (Fig 3C, 3D and 3H). There were no apparent changes in the cell shape between WT and LiCrB25 in the basal and distal position of whorls 1 and 2 organs (S5 Fig in S1 File). The surface area of epidermal cells in the adaxial side of whorls 1 and 2 organs in LiCrB25 was significantly smaller than WT, whereas there was no significant difference in the cell surface area in the abaxial side between WT and LiCrB25 (Table 2). Many papillae consisting of raised epidermal cells were observed on the adaxial surface of the basal position of whorls 1 and 2 organs in WT, whereas only a few papillae were observed in LiCrB25 (S6 Fig in S1 File).

**Table 2:** Surface area of epidermal cells in the adaxial and abaxial sides of whorls 1 and 2 organs of wild-type plants (WT) and a Type I transgenic plants showing a severe morphological alteration (LiCrB25) ^a^.

Floral organ	Plant stain		Basal position			Middle position			Distal position	
		Adaxial side (mm^2^)	Abaxial side (mm^2^)	Adaxial/abaxial sides	Adaxial side (mm^2^)	Abaxial side (mm^2^)	Adaxial/abaxial sides	Adaxial side (mm^2^)	Abaxial side (mm^2^)	Adaxial/abaxial sides
Whorl 1 organ	WT	111.0±3.9	97.3±3.1	1.1±0.1	87.1±4.3	64.4±4.6	1.4±0.0	75.5±3.4	69.1±1.5	1.1±0.0
	LiCrB25	51.7±1.6 *	86.3±8.0 ns.	0.6±0.1 *	56.2±4.9 *	70.7±8.4 ns.	0.8±0.0 *	58.5±1.2 *	68.3±3.5 ns.	0.9±0.1 *
Whorl 2 organ	WT	120.7±2.2	96.2±5.7	1.3±0.1	97.4±7.7	87.2±8.5	1.1±0.0	93.0±2.5	76.9±5.9	1.2±0.1
	LiCrB25	67.3±6.3 *	74.6±7.6 ns.	0.9±0.1 *	52.6±3.2 *	57.3±4.9 *	0.9±0.0 *	53.8±2.4 *	67.7±3.4 ns.	0.8±0.1 *

^a^ Surface area of epidermal cells was measured using the image analysis software ImageJ. Values represent the mean ± standard error of three replicates each consisted of ten epidermal cells.

* and ns. indicate significant and non-significant, respectively, compared with WT (*P*<0.05 by Turkey-Kramer’s test).

### Measurement of the total anthocyanin content in transgenic plants

**Fig 4:**
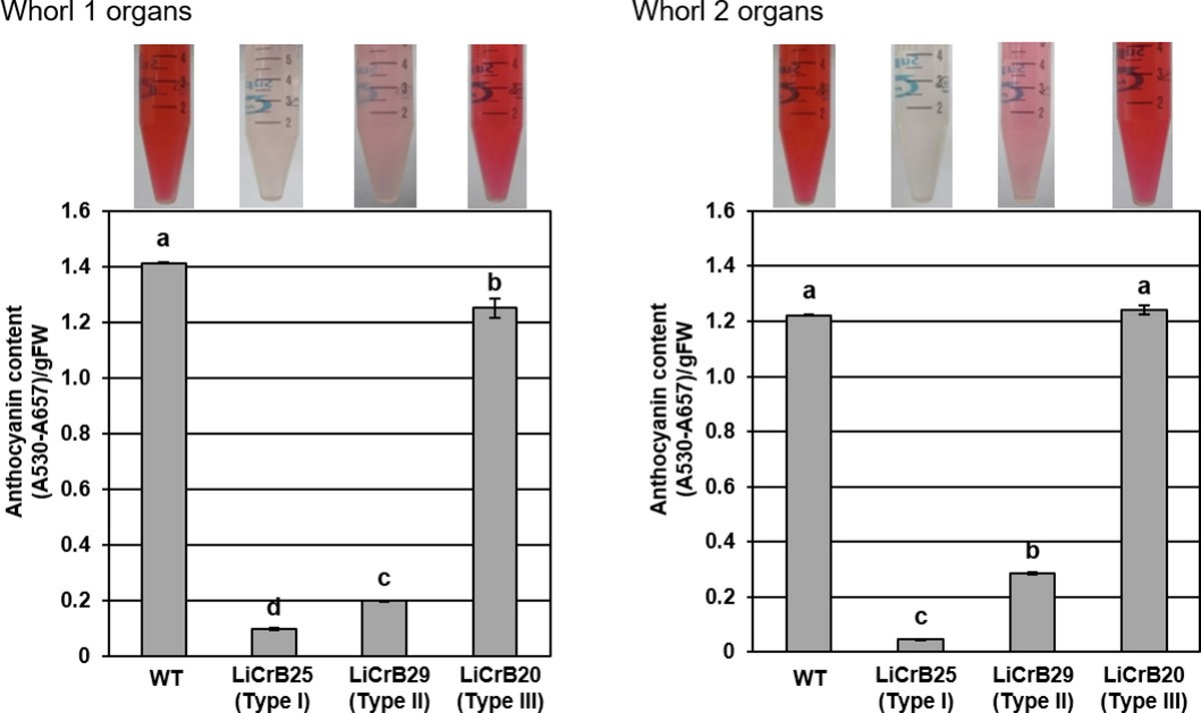
Total anthocyanin content in whorls 1 and 2 organs of wild-type (WT) and transgenic plants (LiCrBs). Type I (LiCrB25), Type II (LiCrB29) and Type III (LiCrB20) transgenic plants showed severe, moderate and no morphological alterations, respectively. Total anthocyanin content on whorls 1 and 2 organs. Values represent the mean ± standard error of triplicates. Values with different letters are significantly different (*P*<0.05 by Turkey-Kramer’s test).

The total anthocyanin contents in whorls 1 and 2 organs of WT and LiCrB strains are shown in Fig 4. Anthocyanin contents in LiCrB25 (Type I) and LiCrB29 (Type II) much decreased compared with WT. In LiCrB20 (Type III), no difference in the anthocyanin content in whorl 2 organs was observed between WT and LiCrB20, although anthocyanin content slightly decreased in whorl 1 organs.

### Expression analysis of endogenous flavonoid biosynthesis-related genes and transgene in transgenic plants

**Fig 5:**
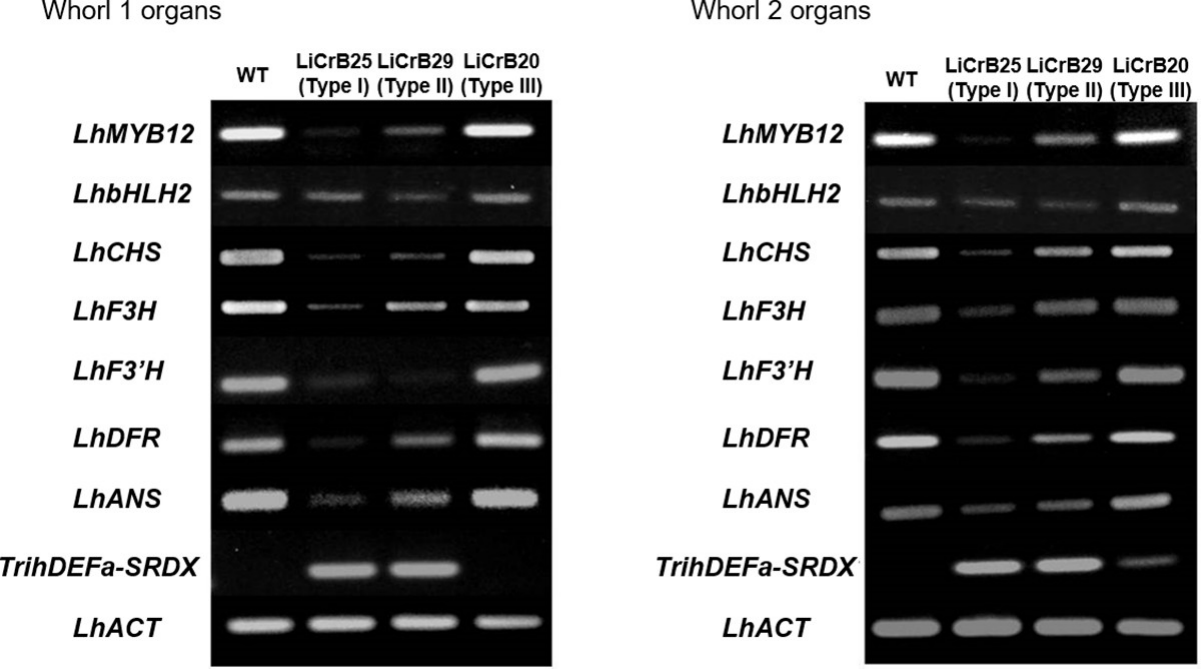
RT-PCR analysis for expression of endogenous flavonoid biosynthesis-related genes (*LhMYB12*, *LhbHLH2*, *LhCHS*, *LhF3H*, *LhF3´H*, *LhDFR* and *LhANS*) and transgene (*TrihDEFa-SRDX*) in whorls 1 and 2 organs of wild-type (WT) and transgenic plants (LiCrBs). Type I (LiCrB25), Type II (LiCrB29) and Type III (LiCrB20) transgenic plants showed severe, moderate and no morphological alterations, respectively. The actin gene of *Lilium* sp. (*LhACT*) was used as an internal control.

The relative expression levels of seven endogenous flavonoid biosynthesis-related genes (*LhMYB12*, *LhbHLH2*, *LhCHS*, *LhF3H*, *LhF3’H*, *LhDFR* and *LhANS*) and transgene (*TrihDEFa-SRDX*) in whorls 1 and 2 organs of WT and LiCrB strains were analyzed by semi-quantitative RT-PCR (Fig 5). Expression levels of all flavonoid biosynthesis-related genes in LiCrB25 (Type I) and LiCrB29 (Type II) greatly decreased compared with WT. On the other hand, LiCrB20 (Type III) showed similar expression levels to WT.

Strong expressions of the transgene were observed in whorls 1 and 2 organs of LiCrB25 and LiCrB29, whereas only a slight expression was observed in whorl 2 organs of LiCrB20. No expression of the transgene was detected in whorls 1 and 2 organs of WT and whorl 1 organs of LiCrB20.

### Expression analysis of endogenous B class genes in transgenic plants

**Fig 6:**
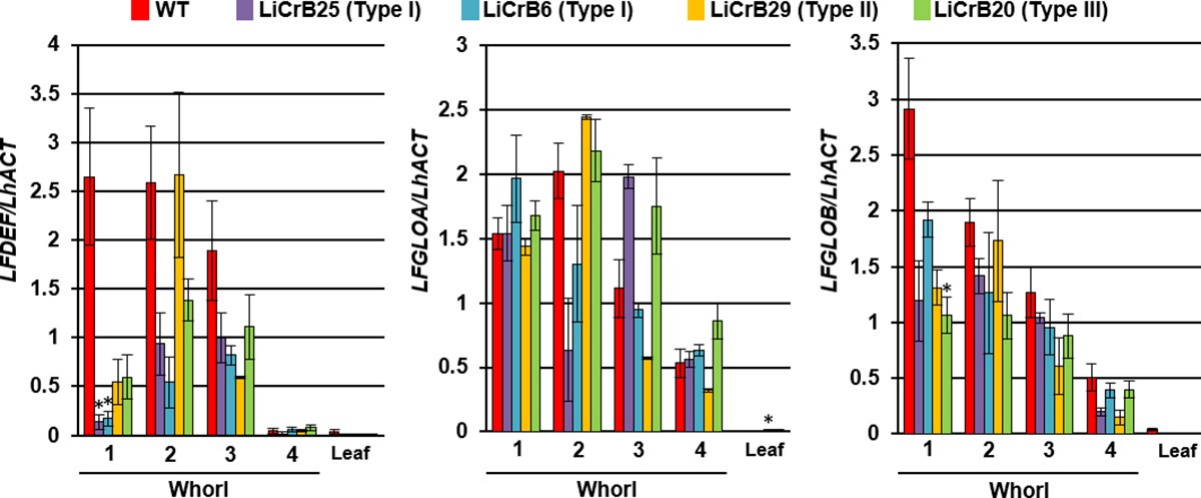
Real-time RT-PCR analysis for expression of endogenous B class genes (*LFDEF*, *LFGLOA* and *LFGLOB*) in floral organs and leaves of wild-type (WT) and transgenic plants (LiCrBs). Type I (LiCrB6 and LiCrB25), Type II (LiCrB29) and Type III (LiCrB20) transgenic plants showed severe, moderate and no morphological alterations, respectively. Relative amounts of transcripts of each gene were normalized to the actin gene of *Lilium* sp. (*LhACT*). Values represent the means ± standard error of triplicates. Asterisk (*) indicates significant difference compared with WT (*P*<0.05 by Turkey-Kramer’s test).

The relative amounts of transcripts of three endogenous B class genes (*LFDEF*, *LFGLOA* and *LFGLOB*) in floral organs and leaves of WT and LiCrB strains were analyzed by real-time RT-PCR (Fig 6). In WT, *LFDEF* transcripts were detected in whorls 1, 2 and 3 organs, whereas transcripts of both *LFGLOA* and *LFGLOB* were detected in whorl 4 organs in addition to whorls 1, 2 and 3 organs. *LFDEF* expression levels in whorls 1, 2 and 3 organs of LiCrB strains largely decreased compared with WT, excepting for whorl 2 organs of LiCrB29 (Type II). Relative amounts of *LFDEF* transcripts in whorl 1 organs were decreased to 6.4, 10.7, 20.5 and 22.5% of WT in LiCrB25 (Type I), LiCrB6 (Type I), LiCrB29 (Type II) and LiCrB20 (Type III), respectively. In addition, relative amounts of *LFDEF* transcripts in whorl 2 organs of LiCrB25 and LiCrB6 decreased to 21.0 and 46.4% of WT, respectively. In contrast to *LFDEF*, no large differences in the relative amount of *LFGLOA* and *LFGLOB* transcripts in floral organs were observed between WT and LiCrB strains.

## Discussion

B class genes have an important role in petaloid organ development. In some plant species with two-layered petaloid tepals, indirect evidence for the modified ABC model has been obtained by detecting B class gene expression in whorl 1 in addition to whorl 2 [14–22]. In the present study, expression of endogenous B class genes (*LFDEF*, *LFGLOA* and *LFGLOB*) were observed in whorl 1 in addition to whorl 2 organs of WT, which is in agreement with the modified ABC model (Fig 6). *LFGLOA* and *TFGLOB* were slightly expressed in whorl 4 organs of WT (Fig 6). Expression of B class genes in whorl 4 organs has also been reported in some monocotyledonous plants producing flowers with two-layered petaloid tepals in whorls 1 and 2 such as *Tricyrtis* sp. [23], *Agapanthus praecox* [16], *Muscari armeniacum* [17, 18] and *Tulipa gesneriana* [14]. Recently, Otani et al. [23] showed direct evidence for the modified ABC model by suppression of B class genes in *Tricyrtis* sp. However, effect of suppressing B function on the floral organ development is unclear in other plant species with two-layered petaloid tepals. In the present study, we produced and characterized transgenic *Lilium* sp. plants with suppressed B function by CRES-T.

Eighteen LiCrB strains carrying the CRES-T construct derived from the B class gene of *Tricyrtis* sp. (*TrihDEFa-SRDX*) were obtained and classified into three types (Type I, II and III) according to the degree of morphological alteration in floral organs. Type I and Type II LiCrB strains showed severe and moderate morphological alterations in floral organs, respectively, while no apparent morphological differences in floral organs were observed in Type III LiCrB strains compared with WT (Fig 1). The degree of morphological alteration in LiCrB strains was positively corelated with the expression level of the transgene *TrihDEFa-SRDX* (Fig 5), while negatively corelated with the expression level of the endogenous B class gene *LFDEF* (Fig 6). B class genes have a positive autoregulatory feedback system that is important for the maintenance of their high expression levels in floral organs [38]. Thus, decreased expression levels of *LFDEF* in LiCrB strains might reflect the degree of inhibition of the autoregulatory feedback system by *TrihDEFa*-*SRDX*. Our results indicate that the LFDEF function may be suppressed by *TrihDEFa-SRDX* expression leading to morphological alteration in floral organs.

In our previous study on CRES-T-mediated suppression of B function in *Tricyrtis* sp., some transgenic plants developed greenish sepaloid tepals in whorls 1 and 2, and pistil-like organs in whorl 3 [23]. However, no such significant alterations were observed in any LiCrB strains. One possible reason for incomplete morphological alteration in floral organs of LiCrB strains is insufficient suppression of B function due to the use of the CRES-T construct derived from a heterologous plant, *Tricyrtis* sp. In transgenic *Pharbitis nil*, suppression of C function by a CRES-T construct derived from its own C class gene resulted in greater morphological effects than that derived from a C class gene of *A*. *thaliana* [39]. Although deduced amino acid sequences of *TFDEF* and *ThirDEFa* show high homology (87.8% of sequence identity and 95.2% of sequence similarity), there are some differences in the sequences of functional domains such as MADS domain and K domain (S7 Fig in S1 File). Further studies are necessary to examine the effect of suppressing B function in *Lilium* sp. by using a CRES-T construct derived from its own B class gene.

In whorls 1 and 2, WT and Type III LiCrB strains developed deep pink-colored tepals, whereas Type I and II LiCrB strains developed pale pink-colored tepals (Fig 1). The amounts of total anthocyanins decreased significantly in whorls 1 and 2 organs of Type I and II LiCrB strains (Fig 4). Expression levels of genes encoding flavonoid biosynthetic enzymes (*LhCHS*, *LhF3H*, *LhF3’H*, *LhDFR* and *LhANS*) also decreased in these organs (Fig 5). In addition, *LhMYB12*, which upregulates transcription of multiple flavonoid biosynthetic enzyme genes in *Lilium* spp. [36], showed lower expression levels in whorls 1 and 2 organs of Type I and II LiCrB strains compared with those of WT and Type III LiCrB strains (Fig 5). Therefore, decreased expression of *LhMYB12* may result in reduced transcription of flavonoid biosynthetic enzyme genes, leading to suppressed anthocyanin synthesis in whorls 1 and 2 organs of Type I and II LiCrB strains. In *Torenia fournieri*, CRES-T-mediated suppression of B function induced reduction of the petaloid identity in whorl 2 organs, in which expression of flavonoid biosynthesis-related gene expression and accumulation of anthocyanins decreased [40]. Thus, pale pink-colored whorls 1 and 2 organs of LiCrB strains in the present study may resulted from reduced petaloid tepal identity by suppression B function.

The size of epidermal cells on the adaxial surface of whorls 1 and 2 organs of Type I and Type II LiCrB strains significantly decreased compared with those of WT, whereas there were no apparent differences in the size of epidermal cells on the abaxial surface among WT, Type I and Type II LiCrB strains (Fig 3; Table 2). Since cell expansion on the adaxial side of tepals is involved in flower opening [41], non-fully-opened flowers of Type I and Type II LiCrB strains may be resulted from an insufficient expansion of epidermal cells on the adaxial side of whorls 1 and 2 organs. A similar observation was obtained in whorls 1 and 2 organs of transgenic *Tricyrtis* sp. with suppressed B function [23]. Non-fully-opening of flowers might be involved in reduction of the petaloid identity and/or conversion of the petaloid identity into the sepaloid identity in whorls 1 and 2 organs.

There were many papillae, which consisted of raised epidermal cells as suggested by Yamagishi and Akagi [42], on the adaxial surface of whorls 1 and 2 organs of WT, whereas only a few papillae were observed in Type I LiCrB strains (Figs 1D and 2; S6 Fig in S1 File). In many *Lilium* species and cultivars, including cv. Acapulco used in the present study, papilla formation on the adaxial surface of whorls 1 and 2 organs is one of the typical traits of petaloid tepals. Although papillae are formed via controlled division of parenchymal and epidermal cells, molecular mechanism of papilla formation in petaloid organs is still unclear. It is possible that cell division patterning of epidermal cells may be changed by reduction of the petaloid tepal identity, reading to decrease in the number of papillae in whorls 1 and 2 organs of Type I LiCrB strains.

In the present study, suppression of B function induced reduction of the petaloid tepal identity of whorls 1 and 2 organs of transgenic *Lilium* sp. This indicates that two-layered petaloid tepals in *Lilium* spp. may be caused by extended expression of B class MADS-box genes in whorl 1 in addition to whorls 2 and 3 proposed as the modified ABC model. The results obtained in the present study in combination with those of our previous study for *Tricyrtis* sp. [23] strongly support the applicability of the modified ABC model in liliaceous plants.

## Supporting information

S1 Raw Images (PDF)

S1 File (PDF)
