# Supplementary figures and images for: Suppression of B function by chimeric repressor gene-silencing technology (CRES-T) reduces the petaloid tepal identity in transgenic Lilium sp
Source: PLoS One. 2020 Aug 3;15(8):e0237176. doi: 10.1371/journal.pone.0237176 (PMC7398511; doi:10.1371/journal.pone.0237176)

**Fig 5. raw data**

**Whorl 1**

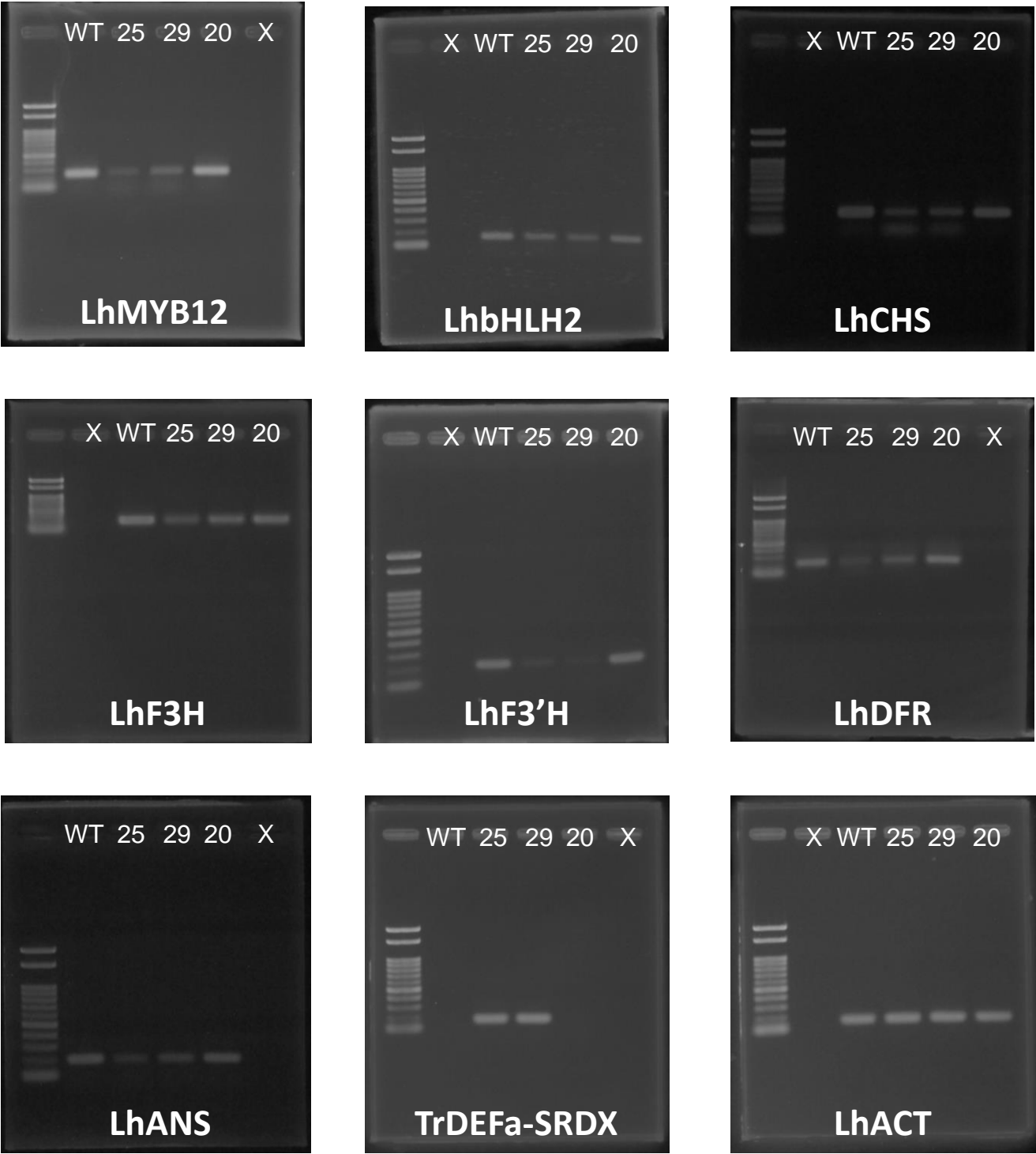

**Fig 5. raw data**

**Whorl 2**

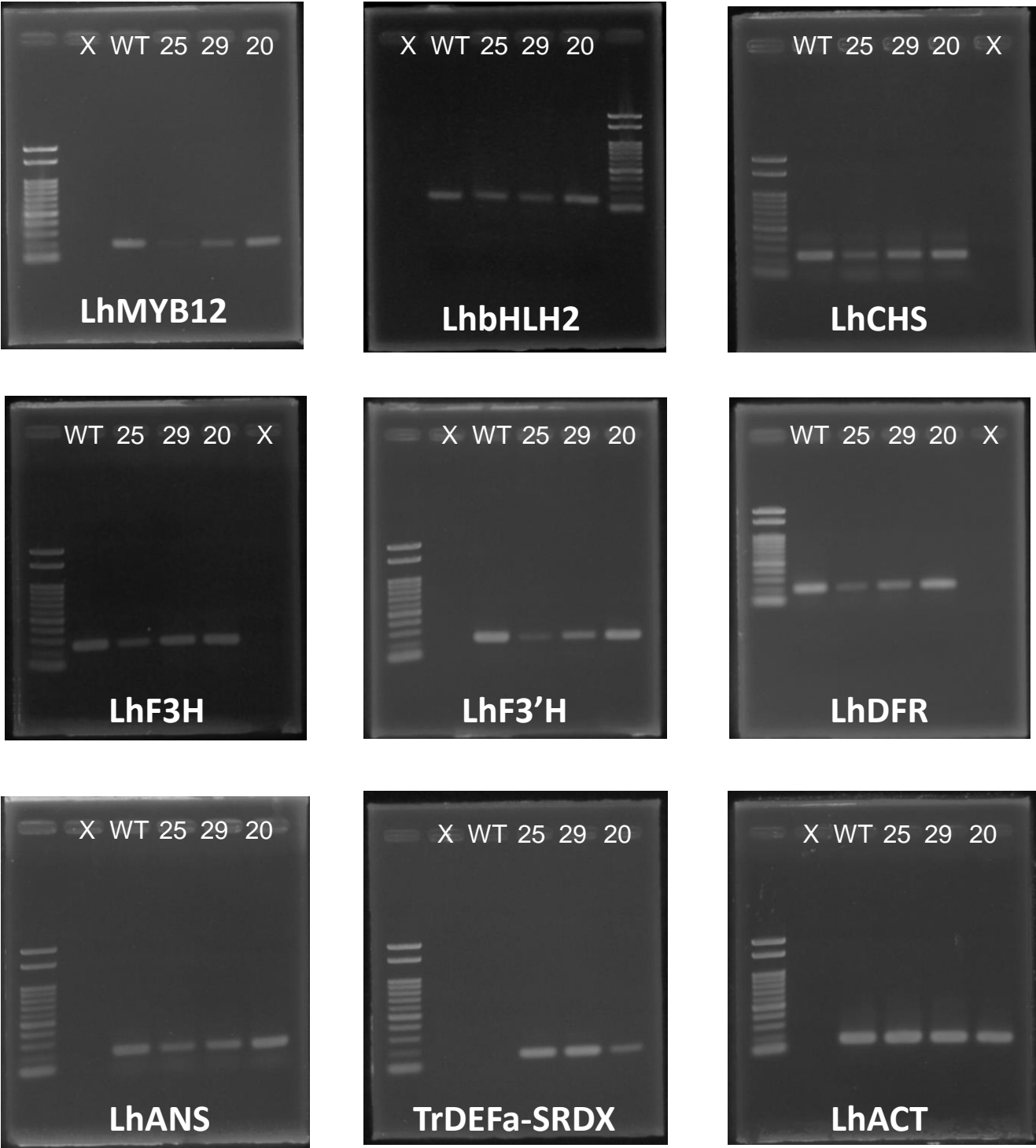

S2 Fig. raw data

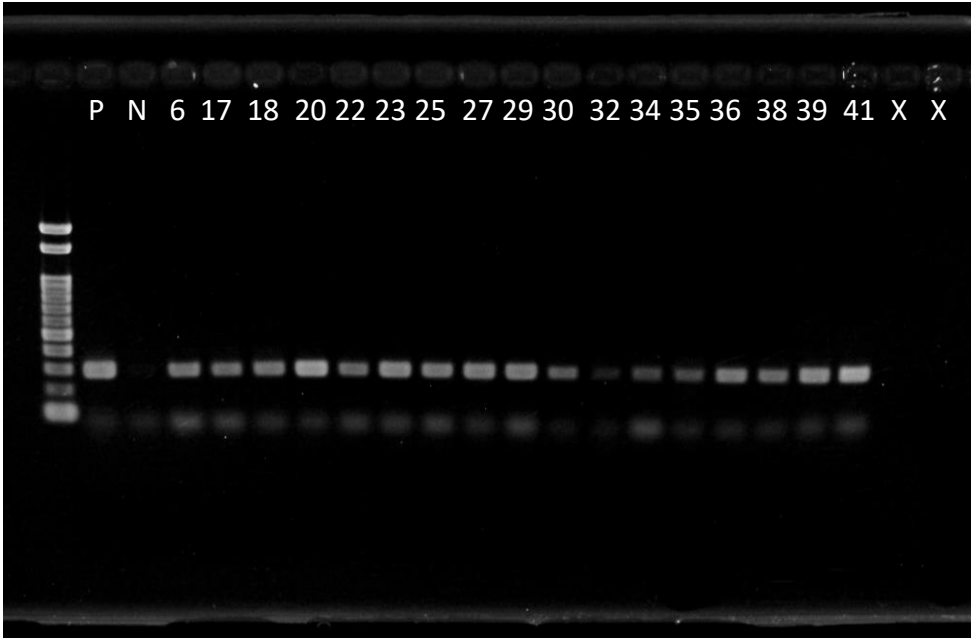

Supplement: S1 Raw Images — (PDF) [file pone.0237176.s001.pdf]
