# Supplementary material for: Suppression of B function by chimeric repressor gene-silencing technology (CRES-T) reduces the petaloid tepal identity in transgenic Lilium sp
Source: PLoS One. 2020 Aug 3;15(8):e0237176. doi: 10.1371/journal.pone.0237176 (PMC7398511; doi:10.1371/journal.pone.0237176)
Supplement: S1 File — (PDF) [file pone.0237176.s002.pdf]

S1 Table. List of accession No. of target genes, primers and PCR conditions used in the present study.

Primer	Sequence (5'→3')	Accession No. of target genes in the GenBank/EMBL/DDBJ databases	PCR condition	Note
LhMYB12 RT-Fw	GAAGAAACAGGGTGAAGCTG	*LhMYB12* (AB534586)	Annealing temperature: 60°C<br>Cycle number: 30	For semi-quantitative RT-PCR
LhMYB12 RT-Rev	CAACTTCGGAATCACTCCAAAG			
LhbHLH2 RT-Fw	TCCAGTTGCCAAACCAGAAC	*LhbHLH2* (AB222076)	Annealing temperature: 60°C<br>Cycle number: 31	
LhbHLH2 RT-Rev	TGGGCAGCTTAGTTCCAAG			
LhCHS RT-Fw	TTCAAGAGGATGTGCGACAAG	*LhCHS* (DQ471950)	Annealing temperature: 60°C<br>Cycle number: 32	
LhCHS RT-Rev	GCTTGTGGTGCAGAAGATGAG			
LhF3H RT-Fw	GTGGCCTCCAAGCAACTAAG	*LhF3H* (AB699160)	Annealing temperature: 60°C<br>Cycle number: 30	
LhF3H RT-Rev	GCATCCAAAACTGGCTTCTC			
LhF3´H RT-Fw	ACGCACGACACAAACTTCAG	*LhF3´H* (AB699161)	Annealing temperature: 60°C<br>Cycle number: 32	
LhF3´H RT-Rev	CAAGTCTCGTGCGAGTATTGC			
LhDFR RT-Fw	TCACAGGAAATGAAGCCCAC	*LhDFR* (AB359185)	Annealing temperature: 60°C<br>Cycle number: 32	
LhDFR RT-Rev	TGGGGGATGGCATATTTAGG			
LhANS RT-Fw	AGTGGTGGTGACCAAGATGC	*LhANS* (AB699166)	Annealing temperature: 60°C<br>Cycle number: 30	
LhANS RT-Rev	TTGGTGAGGAGGAAGGTGAG			
LFDEF RT-Fw	CAAGGAGATCAGCCAGAGGATG	*LFDEF* (AB359185)	Initial denaturation at 95°C for 30 s; 45 cycles of 5 s at 95°C; 30 s at 60°C; and plate reading (detection of	For real-time RT-PCR
LFDEF RT-Rev	CACATGATACTTGCGGTGACG			
LFGLOA RT-Fw	TCAGCAACTGGCAATGGATG	*LFGLOA* (AB359186)		

LFGLOA RT-Rev	ATTGGCTGCACACGAAAGG		fluorescent product)	
LFGLOB RT-Fw	CGGGAGAAGCAGAATGATGTTC	*LFGLOB* (AB359187)		
LFGLOB RT-Rev	CCTCATATTCTCCCCTTGTCAGC			
TrihDEFa RT-Fw	CACTTGCTCTTAGCGAACGGT	*TrihDEFa* (LC080806)		
SRDX RT-Rev	TTAAGCGAAACCCAAACGGAG			
LhActin RT-Fw	GCCATGTATGTTGCAATCCAG	*LhActin* (AB438963)	Semi-quantitative RT-PCR:<br>Annealing temperature: 60°C<br>Cycle number: 30<br><br>Real-time RT-PCR:<br>Initial denaturation at 95°C for 30 s; 45 cycles of 5 s at 95°C; 30 s at 60°C; and plate reading (detection of fluorescent product)	For semi-quantitative RT-PCR and real-time RT-PCR
LhActin RT-Rev	AGCAAGGTCAAGACGAAGGATAG			
htp290-F	GTGCTTTCAGCTTCGATGTAGG		Annealing temperature: 60°C<br>Cycle number: 35	For detecting HPT gene
hpt290-R	GCTCGTCTGGCTAAGATCGG			

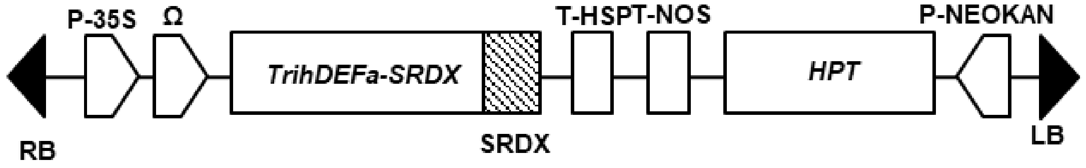

S1 Fig. T-DNA region of the binary vector pBCSH-CrB. *HPT*, hygromycin phosphotransferase gene; LB, left border; P-35S, cauliflower mosaic virus (CaMV) 35S promoter; Ω; the translational enhancer sequence of tobacco mosaic virus; RB, right border; SRDX, ethylene-responsive element binding factor-associated amphiphilic repression (EAR)-motif repression domain; *TrihDEFa*, *DEF* homologous gene of *Tricyrtis* sp.; T-NOS, nopaline synthase terminator; T-HSP, heat shock protein terminator.

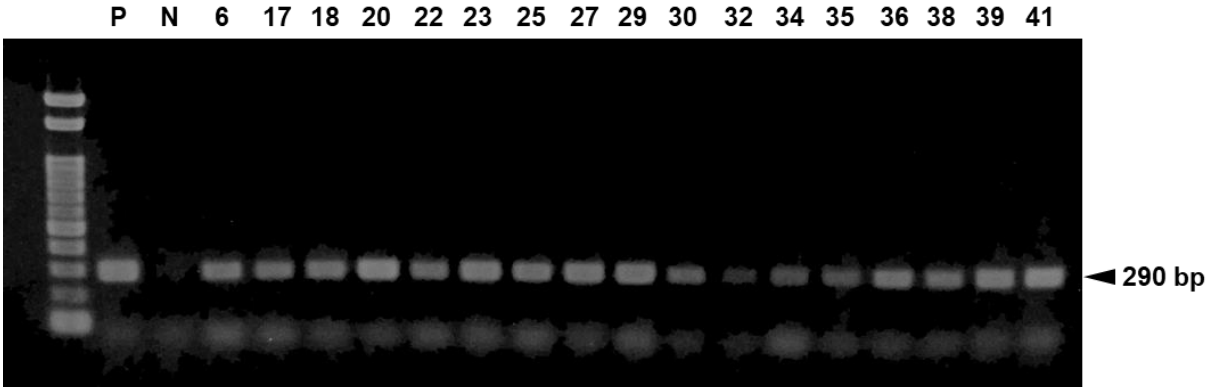

S2 Fig. PCR analysis for detecting *HPT* in putative transgenic plants. Lane P, binary vector pBCSH-CrB as a positive control; lane N, wild-type plant as a negative control; lanes 6–41, independent putative transgenic plants.

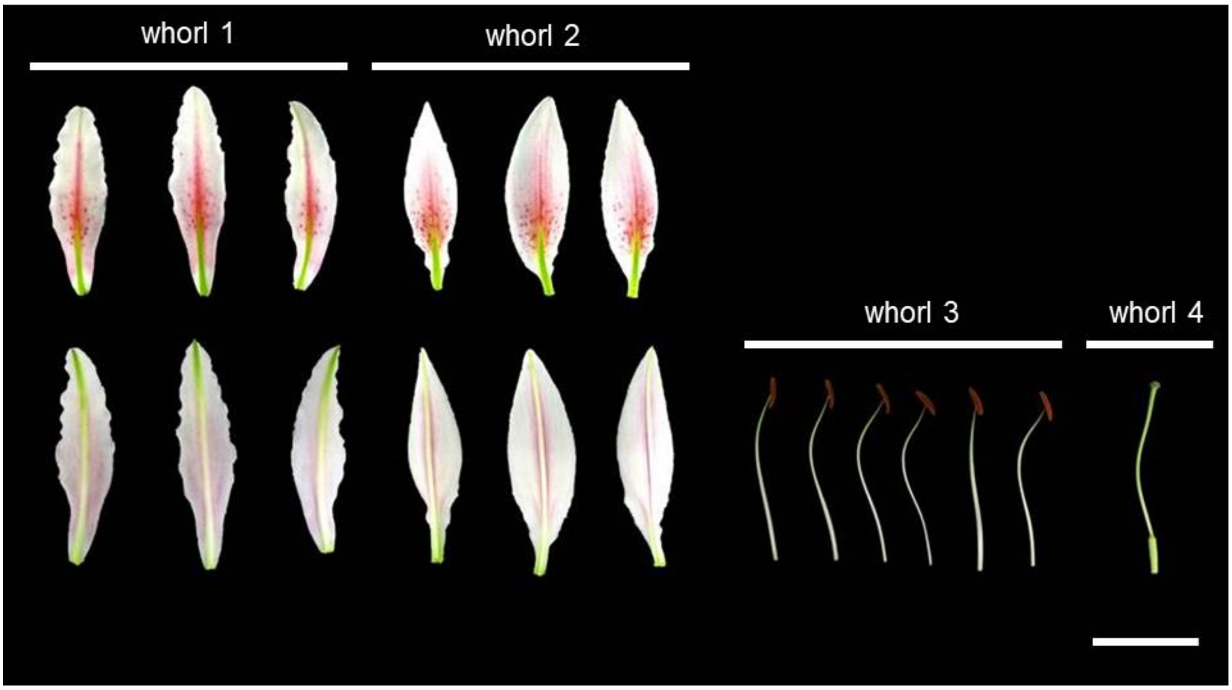

S3 Fig. Floral organs of a Type II transgenic plant (LiCrB29) showing a moderate morphological alteration. Bar = 5 cm.

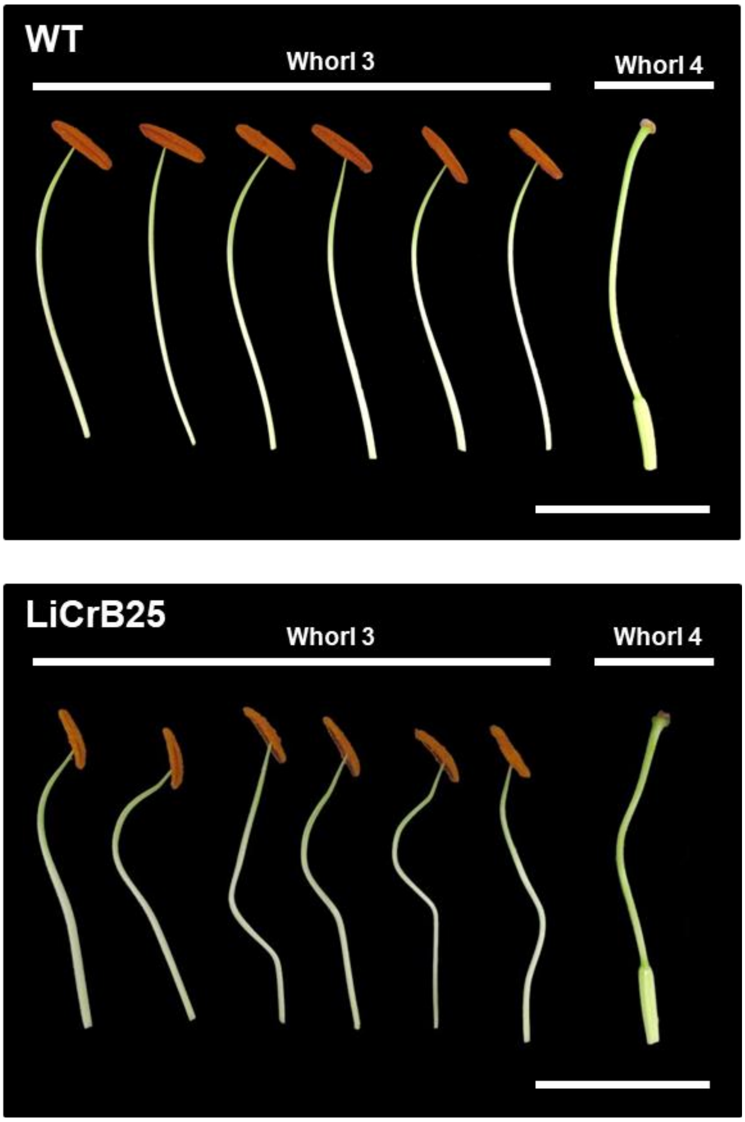

S4 Fig. Whorls 3 and 4 organs of wild-type plants (WT) and a Type I transgenic plant (LiCrB25) showing a severe morphological alteration in whorls 1 and 2 organs. Bar = 5cm.

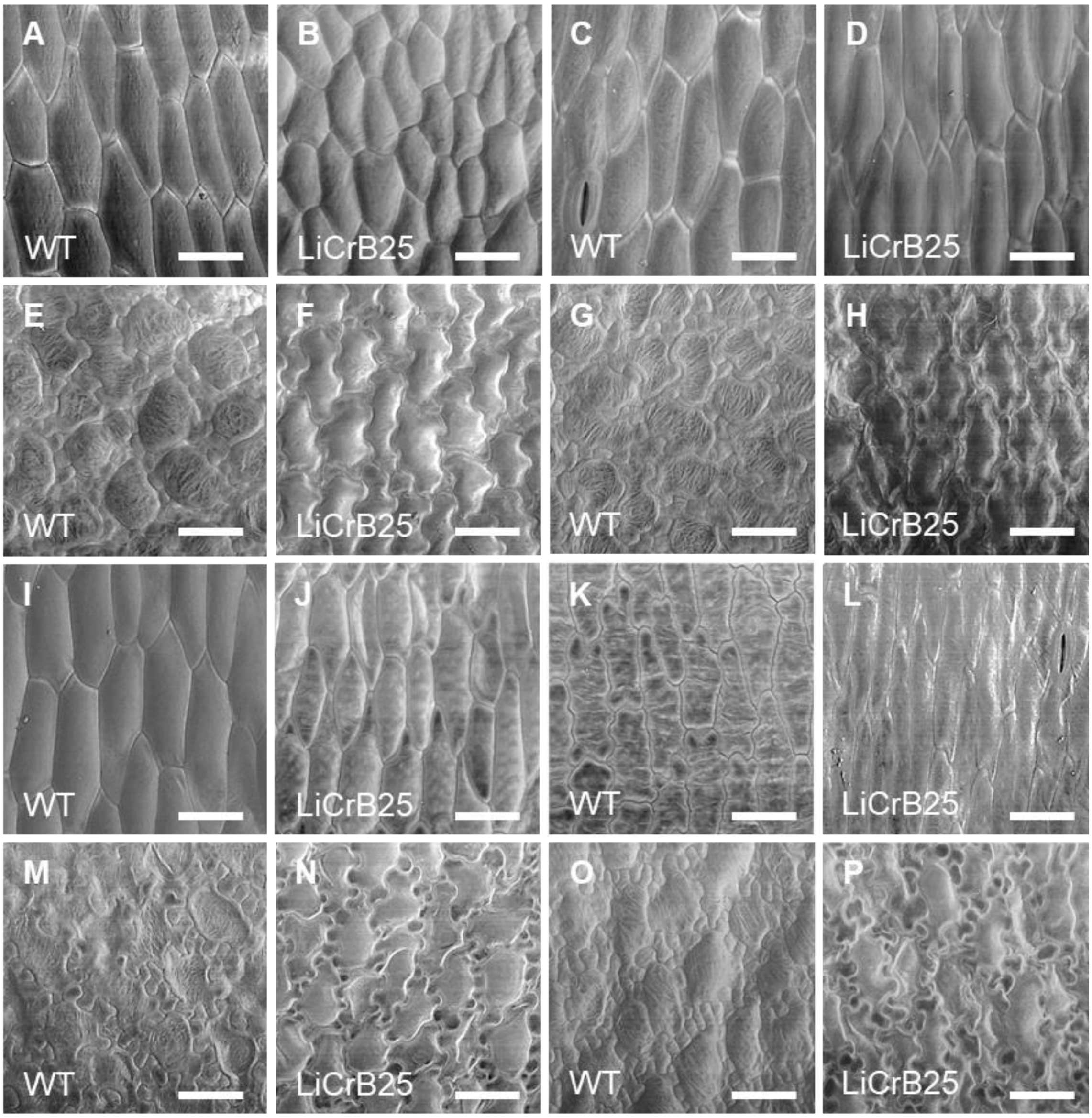

S5 Fig. SEM observation of epidermal cells of whorls 1 and 2 organs of wild-type plants (WT) and a Type I transgenic plant (LiCrB25) showing a severe morphological alteration. (A, B) Adaxial surfaces of the basal position of whorl 1 organs of (A) WT and (B) LiCrB25. (C, D) Abaxial surfaces of the basal position of whorl 1 organs of (C) WT and (D) LiCrB25. (E, F) Adaxial surfaces of the distal position of whorl 1 organs of (E) WT and (F) LiCrB25. (G, H) Abaxial surfaces of the distal position of whorl 1 organs of (G) WT and (H) LiCrB25. (I, J) Adaxial surfaces of the basal position of whorl 2 organs of (I) WT and (J) LiCrB25. (K, L) Abaxial surfaces of the basal position of whorl 2 organs of (K) WT and (L) LiCrB25. (M, N) Adaxial surfaces of the distal position of whorl 2 organs of (N) WT and (M) LiCrB25. (O, P) Abaxial surfaces of the distal position of whorl 2 organs of (O) WT and (P) LiCrB25. Bars = 100 μm.

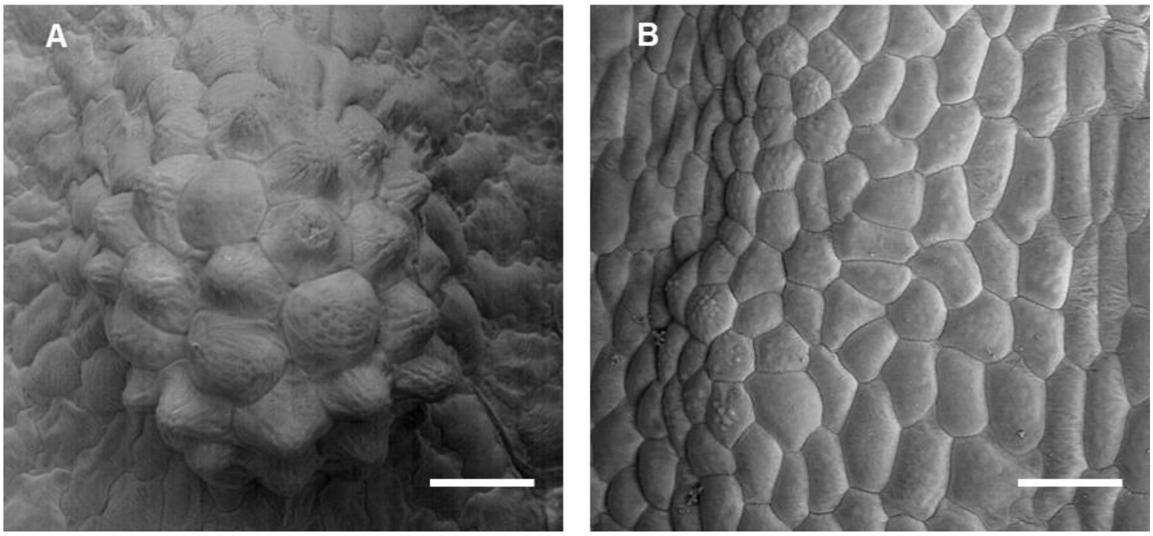

S6 Fig. SEM observations on the adaxial surface of the basal position of whorl 1 organs of (A) wild-type plants and (B) a Type I transgenic plant (LiCrB25) showing a severe morphological alteration. Bar = 100 μm.

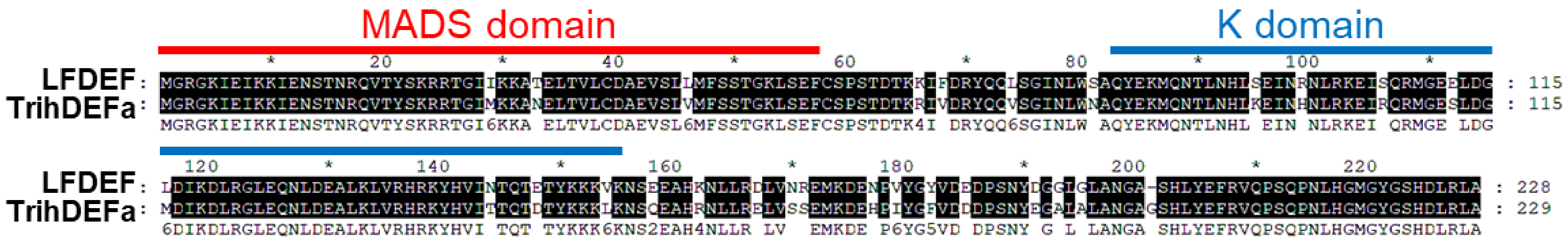

S7 Fig. Alignment of deduced amino acid sequences of *LFDEF* from *Lilium* sp. and *ThirDEFa* from *Tricyrtis* sp. Red and blue lines indicate a putative MADS domain and K domain, respectively.
